# Is cerebral glucose metabolism related to blood–brain barrier dysfunction and intrathecal IgG synthesis in Alzheimer disease? A 18F-FDG PET/CT study: Erratum

**DOI:** 10.1097/MD.0000000000009592

**Published:** 2018-01-05

In the article, “Is cerebral glucose metabolism related to blood–brain barrier dysfunction and intrathecal IgG synthesis in Alzheimer disease? A 18F-FDG PET/CT study”,^[1]^ which appeared in Volume 95, Issue 35 of *Medicine*, Dr. Sofia Toniolo's name appeared incorrectly.
